# Aerobic Exercise Effects on Ocular Dominance Plasticity with a Phase Combination Task in Human Adults

**DOI:** 10.1155/2017/4780876

**Published:** 2017-03-05

**Authors:** Jiawei Zhou, Alexandre Reynaud, Robert F. Hess

**Affiliations:** ^1^School of Ophthalmology and Optometry and Eye Hospital, Wenzhou Medical University, Wenzhou, Zhejiang 325003, China; ^2^McGill Vision Research, Department of Ophthalmology, McGill University, Montreal, QC, Canada H3A 1A1

## Abstract

Several studies have shown that short-term monocular patching can induce ocular dominance plasticity in normal adults, in which the patched eye becomes stronger in binocular viewing. There is a recent study showing that exercise enhances this plasticity effect when assessed with binocular rivalry. We address one question, is this enhancement from exercise a general effect such that it is seen for measures of binocular processing other than that revealed using binocular rivalry? Using a binocular phase combination task in which we directly measure each eye's contribution to the binocularly fused percept, we show no additional effect of exercise after short-term monocular occlusion and argue that the enhancement of ocular dominance plasticity from exercise could not be demonstrated with our approach.

## 1. Introduction

There is ample evidence that the adult brain retains a degree of neural plasticity [1, 2]. In terms of the visual cortex this has been shown in studies on perceptual learning [3–6], noninvasive brain stimulation [7–9], and short-term monocular deprivation [10–14]. Since residual plasticity could be harnessed for therapeutic benefit, it is of interest to know how best to enhance it. A number of rodent models have shown enhancement effects from pharmacological and environmental manipulations. These include the role of donepezil, a centrally acting reversible acetylcholinesterase inhibitor [15], and fluoxetine, a selective serotonin-reuptake inhibitor [16]. Also “environmental enrichment” has been identified as an important factor [17, 18]. This encompasses enhanced motor, sensory, and social activity, of which the motor activity has been assumed to be primal [19]. This is consistent with the finding that visual cortical sensitivity in rodents can be enhanced during motor activity [20] and therefore it has been argued that such enhancement might lead to stronger activity-dependent plasticity [19]. The relationship between visual plasticity and physical activity in rodents is concordant with the generally accepted view that physical activity is beneficial to human adult brain function in general, particularly prefrontal and hippocampal regions [21]. However, it is unclear whether physical activity promotes plasticity in the human visual cortex and in particular the striate cortex, the focus of the present study.

One interesting index of brain plasticity in adult humans is short-term ocular dominance plasticity. This involves the short-term changes that occur in ocular dominance during 2.5 hours of monocular deprivation [10]. This deprivation can be initiated by an opaque patch, a translucent patch [10, 12], or a spatially filtered dichoptic movie [22]. The resultant change in ocular dominance, which is seen in the cellular changes within ocular dominance columns in striate cortex using intrinsic optical imaging [23], is such that the contribution of the previously patched eye to the binocular percept is strengthened while the contribution of the unpatched eye is weakened. Since these neuroplastic effects are reflected through the use of either fusible stimuli, by examining the relative left/right eye contribution to the binocular percept [12, 22, 24], or nonfusible stimuli, by examining the relative left/right eye contributions to binocular rivalry [10, 11, 25], it has been argued that the underlying mechanisms may involve inhibitory interactions at a site before binocular combination [26]. Pertinent to the current debate as to whether physical activity can promote visual cortical plasticity in human adults, a recent study [27] reported that intermittent periods of cycling exercise undertaken during a 2-hour period of monocular patching result in a greater change in ocular dominance, reflecting activity-dependent neuroplasticity. Since this is the first indication that physical activity modulates visual plasticity in the human adult, we wanted to see whether the effects could be generalized to other tasks that would be expected to reflect the same neuroplastic modulation of ocular dominance.

The demonstration [27] of enhanced plasticity as a result of physical activity was shown using a binocular rivalry paradigm, which is one of the two methods that have been used in recent years to quantify ocular dominance plasticity in adult humans. The other approach [12, 22, 24] has involved the use of fusible stimuli by measuring the contribution that each eye makes to the binocularly fused percept and how this eye balance is perturbed by short-term monocular deprivation. Here we use this latter approach to assess what contribution physical activity makes to the neuroplastic modulation of ocular dominance. We use a comparable protocol to that previously reported by Lunghi and Sale [27], in terms of the type of exercise and how it is administered during the 2 hours of monocular occlusion. We assess neuroplastic effects for two levels of exercise in an attempt to define a dose-dependent response. We show no beneficial effect of exercise on ocular dominance plasticity using our binocular combination paradigm for either level of aerobic exercise.

## 2. Methods

### 2.1. Participants

Ten normal adults (mean age: 30.2 ± 1.6 years old; 3 females) with normal or corrected to normal vision participated in this study. Except the first and second authors, all subjects were naive to the purpose of this study. Observers wore their normal optical correction if required. A written informed consent was obtained from each of them before the start of the test. This study complied with the Declaration of Helsinki and was approved by the Institutional Review Boards of Wenzhou Medical University and McGill University. The methods were carried out in accordance with the approved guidelines.

### 2.2. Apparatus

Interocular sensory balance measurements were conducted on a Mac computer using Matlab and PsychToolBox  3.0.9 extensions. The stimuli were dichoptically presented by head mount goggles (eMagin Z800 pro, OLED), with a refresh rate of 60 Hz and a resolution of 800 × 600 in each eye. The mean luminance of OLED goggles was 160 cd/m^2^.

Heartbeat was measured with a Polar H7 heart rate sensor, monitored online, and recorded with the Polar Beat 1.5.2 application running on an Apple iPod Touch G6.

### 2.3. Design

For each observer, the dominant eye was chosen for short-term deprivation. In particular, the dominant eye was deprived by covering with a translucent patch, which transmits light with 80% light transmission but no pattern transmission. The effects of 2-hour monocular patching were accessed by measuring observers' sensory eye dominance before and after the patching period. Three 2-hour patching conditions were studied, which were as follows: (1) resting condition: while the dominant eye was patched, observers were asked to sit quietly to watch a movie; (2) moderate cycling condition: while the dominant eye was patched, observers were asked to watch a movie and do 10 minutes of cycling every 20 minutes. During each 10-minute cycling period, participants were asked to adjust their cycling efforts in order to reach a target heart rate of 60% of their estimated maximum age-related heart rate, calculated as 220 minus the age of the participants, in beats per minute [28]; and (3) hard cycling condition: it is the same as the moderate cycling condition, but with a higher target heart rate (80% of their estimated maximum age-related heart rate).

These three conditions were conducted in a randomized order between subjects on three different days. For each day, observers' ocular dominance was tested before the patching and at 0′, 5′, 10′, 15′, 30′, and 45′ after the completion of the 2 hours of patching. Each test session lasted about 3 minutes.

### 2.4. Procedures

**Figure 1:**
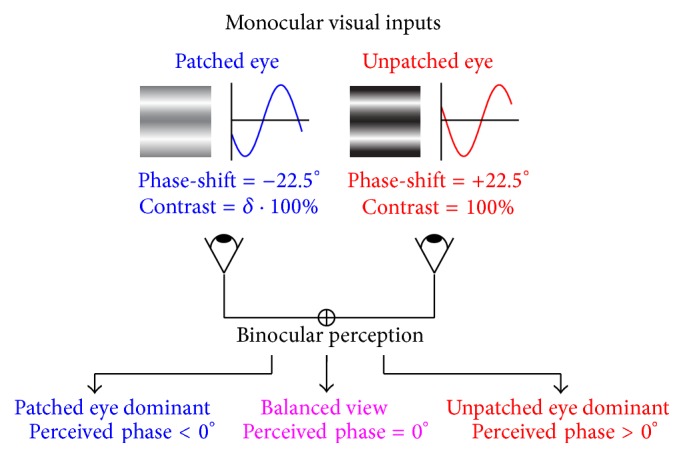
The binocular phase combination paradigm. As illustrated in the figure, two horizontal sine-wave gratings with equal and opposite phase-shift of 22.5° relative to the centre screen were dichoptically presented to the two eyes, and the binocular perceived phase would be 0° when the two eyes are balanced. In our study, we set the phase-shift of the grating to −22.5° in the patched eye and to 22.5° in the unpatched eye. After patching, if the patched eye became stronger, the binocularly perceived phase would be more minus; otherwise, if the patched eye became weaker, the binocularly perceived phase would be more positive.

The change of sensory eye dominance was quantified by a binocular phase combination task, identical to that used previously [12, 24], in which the binocular perceived phase was measured and used as an index of sensory eye dominance. As shown in Figure 1, two horizontal sine-wave gratings (0.3 cycle/°, 6.6° × 6.6°) with equal and opposite phase-shift of 22.5° relative to the centre screen were dichoptically presented to the two eyes; if the patched eye became stronger, the binocularly perceived phase would be more minus; otherwise, if the patched eye became weaker, the binocularly perceived phase would be more positive. For each subject, the contrast of stimuli of their nondominant eye was set as 100% and the contrast of stimuli of their dominant eye was set so that there was equal contribution from each eye to the binocularly fused image (binocularly perceived phase = zero) before the patching. The procedure for measuring perceived phase was similar to that reported in previous studies [29], in which observers were asked to adjust the vertical position of a 1-pixel reference line to indicate the perceived phase of the binocularly perceived horizontal grating, defined by the location of the centre of the dark bar of the grating.

## 3. Results and Discussion

**Figure 2:**
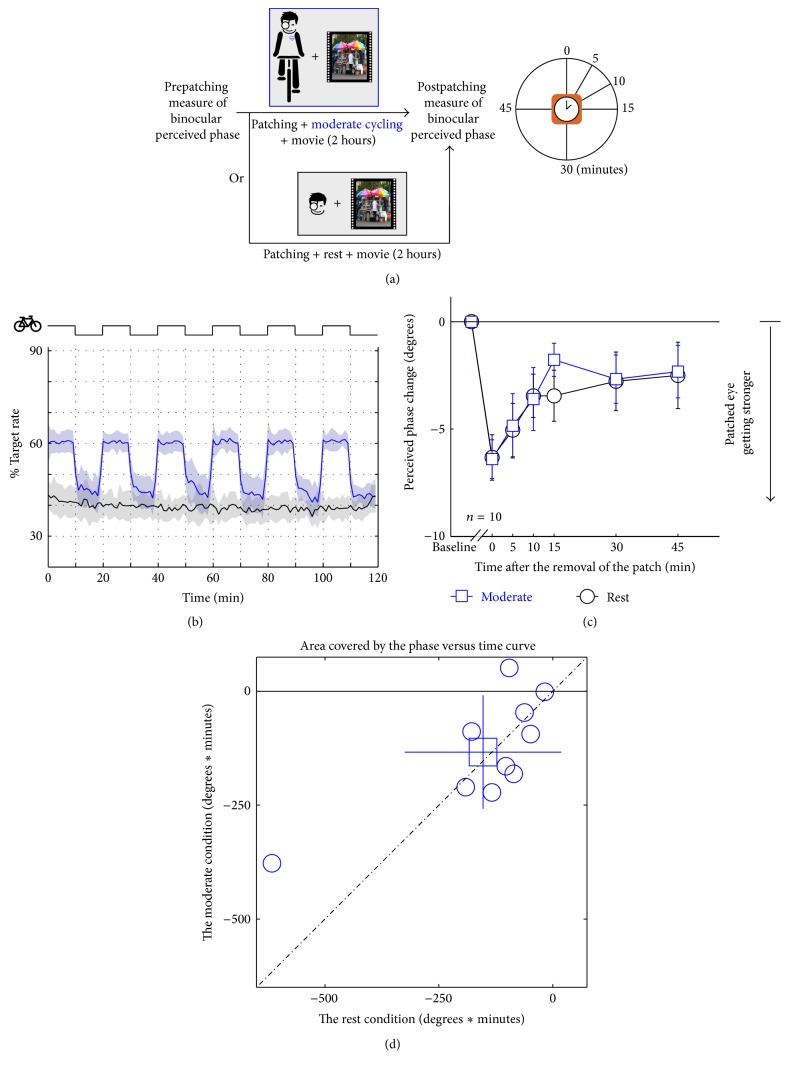
Illustration of the protocol for assessing the effect of moderate exercise on ocular dominance plasticity as a result of short-term monocular deprivation. Subjects (*n* = 10) are monocularly patched while cycling (10 min cycling, 10 min rest) and watching a movie for 2 hours (a). The exercise was intended to raise the heat rate by around 60% of its estimated maximum age-related heart rate (b). The change in ocular dominance as a result of the monocular deprivation is compared for the baseline (resting condition: black lines and open circles) and the exercise condition (blue lines and open squares) (c). The computed areal change (degrees × minutes) is compared for each subject at rest and after exercise; the open square symbol is the group mean ± SD (d).

In Figure 2, the results of moderate exercise on the effects of short-term ocular dominance plasticity are summarized. Ocular dominance is measured using our binocular phase combination task as explained in Methods. Changes in dominance are plotted relative to baseline measurements; a shift in the negative direction indicates that the previously patched eye is more dominant. The at-rest results (Figure 2(c)) for a group of healthy young observers (age 30.20 ± 1.55 years) are displayed in black with open circles. The plasticity of ocular dominance is seen to last for about 30 minutes after removal of the monocular occluder; this is consistent with all our previous studies [12, 22]. The exercise involved cycling on an exercise bike for 10-minute periods every 20 minutes during the deprivation period. Subjects watched a movie of their choice for the 2-hour period during which they were monocularly deprived. The exercise was designed to increase the heart rate by around 60% of its estimated maximum age-related heart rate. The heart rate was monitored and the average heart rates before and after exercise are shown in Figure 2(b). Subjects found this degree of exercise significant but manageable. The results showing the effect of exercise on ocular dominance plasticity are plotted in blue with open square symbols. No significant effect of exercise was found compared to the at-rest baseline. A repeated-measures within-subject Analysis of Variance (ANOVA) also showed that the perceived phase change was significantly varied by time (*F*(5,45) = 10.16, *p* < 0.001), but not significantly different between the exercise and no exercise conditions (*F*(1,9) = 0.21, *p* = 0.66); the interaction of these two factors was also not significant (*F*(5,45) = 0.39, *p* = 0.85). In Figure 2(d), the computed areal change (degrees × minutes) is compared for each subject at rest and after exercise. The average ratio between the areal change at the moderate condition and that at the rest condition was 0.981 ± 0.856 (mean ± SD), which was not significantly different with 1: *t*(9) = −0.07, *p* = 0.95 (2-tailed one sample* t*-test).

**Figure 3:**
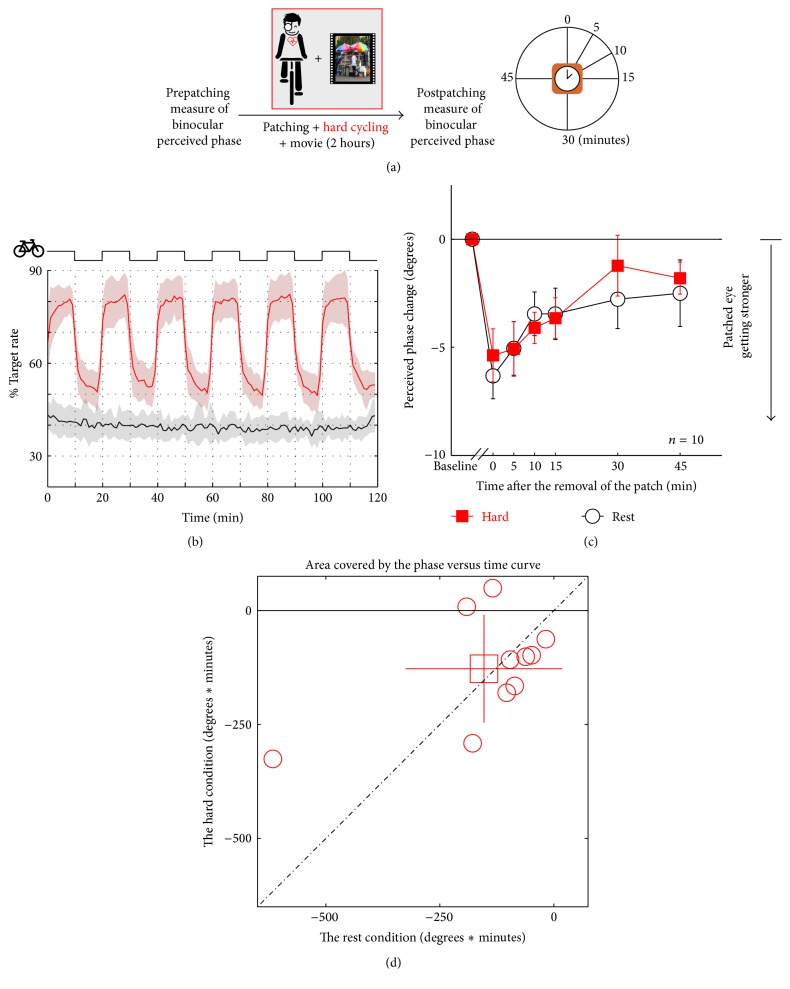
Illustration of the protocol for assessing the effect of severe exercise on ocular dominance plasticity as a result of short-term monocular deprivation. Subjects (*n* = 10) are monocularly patched while cycling (10 min cycling, 10 min rest) and watching a movie for 2 hours (a). The exercise raises the heat rate by around 80% of its estimated maximum age-related heart rate (b). The change in ocular dominance as a result of the monocular deprivation is compared for the baseline (resting condition: black lines/open circles) and exercise condition (red lines/filled squares) (c). The computed areal change (degrees × minutes) is compared for each subject at rest and after exercise; the open square symbol is the group mean ± SD (d).

In Figure 3, results are shown for the same protocol repeated but where the exercise was more strenuous. Now the exercise was increased so that the heart rate was increased by around 80% of its estimated maximum age-related heart rate (Figure 3(b)). Subjects considered this a demanding exercise routine. Ocular dominance plasticity measured before and after this heavier exercise is shown in Figure 3(c) as black lines/open circles and red lines/filled squares, respectively. No influence of exercise on ocular dominance plasticity was observed. A repeated-measures within-subject ANOVA also showed that the perceived phase change was significantly varied by time (*F*(5,45) = 8.01, *p* < 0.001), but not significantly different between the exercise and no exercise conditions (*F*(1,9) = 0.21, *p* = 0.66); the interaction of these two factors was also not significant (*F*(5,45) = 0.71, *p* = 0.62). In Figure 3(d), the computed areal change (degrees × minutes) is compared for each subject at rest and after exercise. The average ratio between the areal change at the hard condition and that at the rest condition was 1.383 ± 1.144 (mean ± SD), which was not significantly different with 1: *t*(9) = 1.06, *p* = 0.32 (2-tailed one sample* t*-test).

When one eye is deprived of spatial contrast for a period of around 2 hours, there is an observable change in ocular dominance that lasts for about 30 minutes. The previously deprived eye becomes more dominant and the nondeprived eye becomes less dominant. Originally this was demonstrated using binocular rivalry as an index of ocular dominance [10] and later shown using fusible stimuli, by determining the relative left/right eye contribution to the binocularly fused percept [12, 22].

The recent report that exercise enhances this plasticity effect when assessed with binocular rivalry [27] is not generalized to the use of fusible stimuli used in the present study. It is unclear why such an exercise enhancement would not be reflected in our measurements. There are two obvious possibilities; the first is that the approach we use lacks sensitivity, and the second is that our sample size is too small. Concerning the first issue, the approach we use has been shown to be sensitive in that it can reveal much smaller changes in dominance produced by much subtler forms of deprivation that achieved by translucent occlusion. These subtle changes in dominance are the result of monocular spatial filtering of dichoptically presented videos [22]. It is unlikely to be due to ceiling effects (i.e., saturation), as we have previously shown [24], using the same technique, that both the magnitude and the duration of the dominance change can be larger in some humans with amblyopia. Concerning the sample size, it should be pointed out that any trends we find from exercise are in the opposite direction, so we would have to assume that all our subjects were outliers to entertain an explanation based on sample size. Another way of addressing this issue is to calculate, from the effect size previously reported by Lunghi and Sale [27] using binocular rivalry, given our measurement variance, how many subjects we should need to achieve a power greater than 80%. The areal analysis shown in panel (d) of Figures 2 and 3 allows a comparison with Lunghi and Sale's [27] results (see Supplemental Information for full analysis in Supplementary Material available online at https://doi.org/10.1155/2017/4780876). For our moderate exercise conditions we calculate that only the results from 2 subjects should be sufficient and for the hard exercise condition only 3 subjects are necessary. The 10 subjects we tested should certainly have been sufficient. A remaining possibility is that these two methods (binocular rivalry and binocular combination) reflect very different neural processes. The approach that we have taken involves the direct estimation of each eye's contribution to the binocularly fused percept. It can be modeled in terms of the standard contrast-gain control model [30] that describes the excitatory and inhibitory interactions that are limited to the striate cortex/LGN circuit [31, 32]. Binocular rivalry arises from the competition between neurons in the LGN and in V1 [33–36] and reflects amongst other things the contralateral inhibitory interactions that are known to occur prior to binocular combination. Its neural circuitry extends well beyond the striate visual cortex as it is contour dependent and potentially involves neural competition at multiple levels of the visual pathways beyond the striate cortex [25, 37, 38]. Neural correlates of the perceptual fluctuations have been found in the parietal cortex and the frontal cortex [38, 39], which explains the high susceptibility of binocular rivalry to attention [40]. It is possible, based on the known effects of exercise on prefrontal and hippocampal regions [21], that the exercise-dependent effects for binocular rivalry could involve a top-down influence of a more general, nonvisual nature [41]. All that we can say at the moment is that the enhancement of dominance plasticity due to excise is not reflected in all measures.

## 4. Conclusions

We conclude that any effects of exercise on ocular dominance plasticity are not revealed using our binocular combination approach.

## Supplementary Material

Sample size calculations. Estimation of the required sample size to obtain a significant effect of the aerobic exercise.
