# Supplementary material for: Aerobic Exercise Effects on Ocular Dominance Plasticity with a Phase Combination Task in Human Adults
Source: Neural Plast. 2017 Mar 5;2017:4780876. doi: 10.1155/2017/4780876 (PMC5357532; doi:10.1155/2017/4780876)
Supplement: Supplementary file 1 — Sample size calculations. Estimation of the required sample size to obtain a significant effect of the aerobic exercise. [file 4780876.f1.pdf]

Supplemental Information

Sample size calculations

To be able to compare Lunghi & Sale's (Lunghi and Sale 2015) approach with ours (Binocular rivalry and Binocular combination) we have now provided in each of our results figures an analysis of our results in terms of area (phase x time) so it can be compared with Lunghi & Sale's (eye rivalry x time).

The ratios between the area of the Physical activity and the inactive control in our two experiments are:
1. moderate/rest: 0.981 ± 0.856 (mean ± SD), which was not significantly different with 1: t(9)=-0.07, *p* = 0.95 (2-tailed one sample t-test);
2. hard/rest: 1.383 ± 1.144 (mean ± SD), which was not significantly different with 1: t(9)=1.06, *p* = 0.32 (2-tailed one sample t-test).

The performance increase in Lunghi & Sale's cycling paper (Lunghi and Sale 2015) could be calculated from the relative difference between the area under the fitted curves (AUC) in the cycling and resting conditions in their figure 1B (equation (S1), parameters a and b of the fits and the baseline ratio could be found in their supplementary):

$$AUC=\sum_t 1+\left(\frac{a}{\left(\log\left(t+1\right)\right)}\right)^b \quad \text{(S1)}$$

Then the relative difference between their cycling and resting state is 2.1784 (between 1 minute and 45 minute).

To calculate the sample size for matched samples we would need to obtain the same margin of error E=2.1784, we use the equation (S2):

$$n=\left(\frac{\left(Z_\beta+Z_{\alpha/2}\right)\sigma_d}{E}\right)^2 \quad \text{(S2)}$$

where Z_β = 0.84 corresponds to the selected 80% power, $Z_{\alpha/2}$ = 1.96 corresponds to the 2-tailed significance level at $\alpha=0.05$ and σ_d is the standard deviation of the matched relative differences between our cycling and resting conditions.

So, with σ_d = 0.856 in the moderate condition and σ_d = 1.144 in the hard condition, we obtain a desired sample size n = 1.21 and n = 2.16 respectively for the moderate and hard cycling condition.
